# Associations Between Subjective Symptoms and Serum Immunoglobulin E Levels During Asian Dust Events

**DOI:** 10.3390/ijerph110807636

**Published:** 2014-07-29

**Authors:** Shinji Otani, Kazunari Onishi, Haosheng Mu, Takenobu Hosoda, Youichi Kurozawa, Masahide Ikeguchi

**Affiliations:** 1 Division of Surgical Oncology, Faculty of Medicine, Tottori University, Yonago 683-8504, Japan; E-Mail: masaike@med.tottori-u.ac.jp; 2 Arid Land Research Center, Tottori University, Tottori 680-0001, Japan; 3 Division of Health Administration and Promotion, Faculty of Medicine, Tottori University, Yonago 683-8503, Japan; E-Mails: issey@med.tottori-u.ac.jp (K.O.); muhs@med.tottori-u.ac.jp (H.M.); thosoda@med.tottori-u.ac.jp (T.H.); kurozawa@med.tottori-u.ac.jp (Y.K.)

**Keywords:** Asian dust, type 1 allergic reaction, immunoglobulin E

## Abstract

Asian dust is a seasonal meteorological phenomenon caused by the displacement of atmospheric pollutants from the Mongolian and Chinese deserts. Although the frequency of Asian dust events and atmospheric dust levels have steadily increased in the eastern Asia region, the effects on human health remain poorly understood. In the present study, the impact of Asian dust on human health was determined in terms of allergic reactions. A total of 25 healthy volunteers were tested for a relationship between serum immunoglobulin E (IgE) levels and subjective symptoms during a 3-day Asian dust event recorded in April 2012. They filled daily questionnaires on the severity of nasal, pharyngeal, ocular, respiratory, and skin symptoms by a self-administered visual analog scale. Serum levels of non-specific IgE and 33 allergen-specific IgE molecules were analyzed. Spearman rank-correlation analysis revealed significant positive associations between nasal symptom scores and 2 microbial-specific IgE levels (*Penicillium* and *Cladosporium*). Microbes migrate vast distances during Asian dust events by attaching themselves to dust particles. Therefore, some of these symptoms may be associated with type 1 allergic reactions to certain type of microbes.

## 1. Introduction

Asian dust events are massive meteorological phenomena during which fine particles from Taklamakan Desert, Gobi Desert, and Loess Plateau in inland China are blown into the atmosphere and carried by westerly winds into northeast Asia. There is a steady increase in the frequency of these events and atmospheric dust levels in East Asia because of industrial pollutants and active desertification in China [1,2]. Nonetheless, the impact of Asian dust on human health remains poorly understood. 

We previously reported an association between skin symptoms and Asian dust events [3,4]. Subjects with a tendency of metal allergy are more susceptible to develop skin symptoms during Asian dust events [5]. Moreover, organic agents adhering to Asian dust particles are involved in the allergic reactions [6,7]. Immunoglobulin E (IgE) plays an essential role in type 1 hypersensitivity associated with allergic diseases such as asthma, allergic rhinitis, and food allergy. To evaluate the effects of Asian dust on human health in terms of allergic reaction, we investigated the relationship between serum IgE levels and subjective symptoms during a 3-day Asian dust event.

## 2. Experimental Section 

### 2.1. Subjects 

The study subjects were 25 healthy volunteers. They lived in Yonago, which is among the cities with the highest annual frequency of Asian dust events in western Japan. The volunteers were selected from students and staff of the Tottori University Faculty of Medicine, and provided informed consent. This study was approved by the Ethics Committee of the Faculty of Medicine, Tottori University.

### 2.2. Health Survey on the Effects of Asian Dust Events

Symptom scores were measured daily from 19 to 30 April 2012 to monitor allergic responses before, during, and after the Asian dust event (23–25 April 2012, visibility < 10 km; Japan Meteorological Agency). All participants completed questionnaires covering nasal (sneezing, nasal discharge, congestion, and itching), pharyngeal (sore throat and scratchy throat), ocular (itching, lacrimation, hyperemia, and bleary eyes), respiratory (cough, sputum, breathlessness, chest pain, chest discomfort, and dyspnea), and skin (itching, eczema, pain, and reddish skin) symptoms. The participants recorded daily symptoms by self-administered visual analog scale (VAS; 0–5 points), and the score was calculated as the average number of points per symptom per person. Asian dust-related symptom scores were obtained on 24–26 April: one day behind Asian dust days, because the effect of Asian dust on subjective symptoms is perceived to become clear at second day after dust exposure in previous study [4].

### 2.3. IgE Measurements 

Serum levels of non-specific IgE and 33 allergen-specific IgEs were measured after the Asian dust events to account for delayed peak allergic response using a chemiluminescent enzyme immunoassay (MAST33, SRL Co., Ltd., Tokyo, Japan). The allergen-specific IgEs included food allergens (buckwheat, wheat, peanut, soybean, rice, tuna, salmon, shrimp, crab, cheddar cheese, milk, beef, chicken, and egg), pollen allergens (timothy grass, vernal grass, *Dactylis glomerata*, *Ambrosia artemisiifolia*, artemisia, cedar, hinoki cypress, alder, and white birch), environmental allergens (house dust mite, house dust, cat desquamated skin, and dog desquamated skin), microbial allergens (*Penicillium*, *Cladosporium*, *Candida*, *Alternaria*, and *Aspergillus*), and latex.

### 2.4. Statistical Analysis

All data analyses were performed using SPSS 21.0 for Windows (IBM, Armonk, NY, USA). The relationships between symptom scores and allergy indicators were assessed using Spearman rank-correlation, with a significance level of 5%. 

## 3. Results and Discussion

### 3.1. Results 

**Figure 1:**
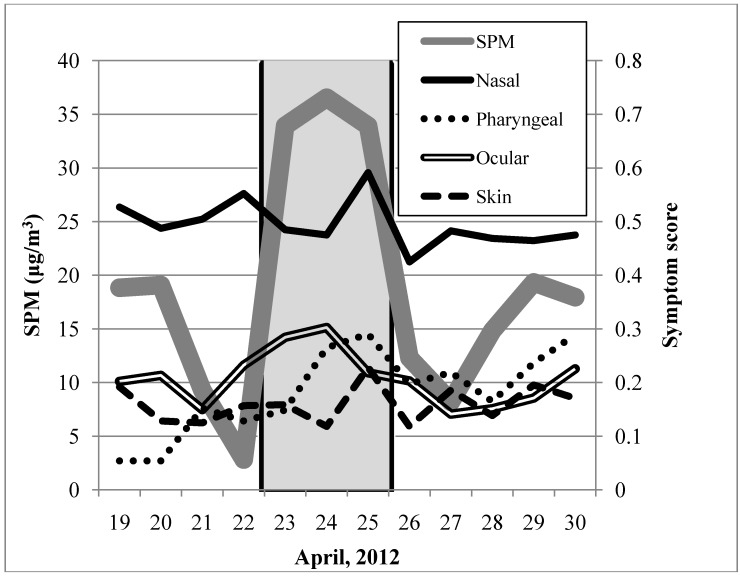
Time-course measurements of SPM levels and symptom scores from 19 to 30 April 2012. The gray zone indicates the Asian dust event.

During 3 days of an Asian dust event, the average level of suspended particulate matter (SPM, a diameter of ≤10 μm) obtained from Tottori Prefectural Institute of Public Health and Environmental Science was 34.3 μg/m^3^ in Yonago (average of SPM levels of non-Asian dust days during the study period was 13.6 μg/m^3^). Asian dust-related symptom scores were as follows: nasal, 0.42; pharyngeal, 0.23; ocular, 0.15; respiratory, 0.00; and skin, 0.18. In addition, time-course analysis of SPM levels and symptom scores (except for respiratory) was conducted from 19 to 30 April 2012 (Figure 1). 

The average serum level of each allergen-specific IgE (lumicount: ≤1.40, negative; 1.40–2.77, false-positive; ≥2.78, positive) was as follows: buckwheat, 0.27; wheat, 0.84; peanut, 0.40; soybean, 0.59; rice, 0.44; tuna, 0.41; salmon, 0.44; shrimp, 0.43; crab, 2.67; cheddar cheese, 0.83; milk, 1.08; beef, 0.65; chicken, 0.10; egg, 0.84; timothy grass, 5.02; vernal grass, 8.19; *Dactylis glomerata*, 2.43; *Ambrosia artemisiifolia*, 0.22; artemisia, 1.35; cedar, 41.59; hinoki cypress, 5.28; alder, 0.28; white birch, 0.72; house dust mite, 11.60; house dust, 11.76; cat desquamated skin, 1.88; dog desquamated skin, 0.61; *Penicillium*, 1.04; *Cladosporium*, 0.42; *Candida*, 0.73; *Alternaria*, 0.47; *Aspergillus*, 0.89; latex, 0.28.

The correlation coefficients between the symptom scores and serum levels of IgE were significant for two fungal allergens, namely *Penicillium* and *Cladosporium* allergens (*r* = 0.477 and 0.519, respectively). The Spearman rank-correlation coefficients between the symptom scores, except for respiratory and serum levels of fungal-specific IgE, are shown Table 1.

**Table 1:** Spearman rank-correlation coefficients between symptom scores and IgE serum levels.

Symptom Score	Total	Nasal	Pharyngeal	Ocular	Skin
Non-specific IgE	0.000	−0.144	−0.088	0.020	0.261
*Penicillium*	0.266	0.477 ^*^	−0.021	−0.207	0.146
*Cladosporium*	0.201	0.519 ^**^	−0.146	−0.209	−0.005
*Candida*	0.267	0.374	−0.078	−0.169	0.281
*Alternaria*	−0.054	0.195	−0.271	−0.355	0.073
*Aspergillus*	0.261	0.257	0.215	−0.195	0.184

^*^p = 0.016, ^**^p = 0.008

### 3.2. Discussion 

The present study shows that nasal symptom scores collected during Asian dust events are primarily associated with IgE levels of microbial allergens (*i.e.*, *Penicillium* and *Cladosporium*) even though the highest IgE levels were reported for pollen and environmental allergens. Our results are consistent with earlier reports suggesting a relationship between organic agents adhering to Asian dust particles and allergic reactions [6,7]. Previous studies showed that Asian dust events were accompanied by an increase in daily hospital admissions and clinic visits or worsening of symptoms for allergic diseases such as asthma and allergic rhinitis [8,9]. Type 1 hypersensitivity plays a central role in asthma and allergic rhinitis. Therefore, our results suggest that some symptoms associated with Asian dust events may involve type 1 allergic reactions.

Recent studies have showed that microbes, such as bacteria and fungi, migrate vast distances during Asian dust events by attaching themselves to dust particles [10,11]. In Korea, springtime air contains a large variety of fungi and potentially high levels of fungal allergens including *Penicillium* [11]. In Japan, fungal attachment to dust particles has not been confirmed. However, Yamaguchi *et al.* identified the possibility of bacterial attachment to aeolian dust particles and global migration during dust events [10]. Additionally, the present study demonstrates a significant association between IgE levels of microbial allergens (*i.e.*, *Penicillium* and *Cladosporium*) and nasal symptom scores. Together, these data suggest that Asian dust events may trigger type 1 hypersensitivity to fungal allergens.

Cedar pollinosis is a major public health issue in spring, and allergy to house dust is a common condition in Japan. In our study, the serum levels of IgE for cedar, house dust mite, and house dust were very high (41.59, 11.60, and 11.76 lumicount, respectively) compared with those for other allergens. Although these allergens may be problematic for the Japanese population, the present study suggests that they are not related to the symptoms developing during Asian dust events.

The main limitation of this study was the possible interference by other environmental factors such as pollen dispersal and local air pollution. However, pollen dispersal in Yonago was particularly low in April 2012 than in previous months. Moreover, there is no major source of air pollution in this area, aside from a polluting factory and motor vehicles. Finally, we could not assess the impact of Asian dust events by multivariate analysis due to an insufficient sample size. Therefore, large-scale studies including multiple environmental data and confirmation of fungal attachment to Asian dust particles in the survey area are required to confirm the relationships identified in the present study.

## 4. Conclusions 

This is the first report demonstrating an association between nasal symptoms and fungal allergen IgE levels during Asian dust events. This study also emphasizes the need to consider type 1 hypersensitivity as a significant health risk during Asian dust events.
